# A transferrin receptor’s guide to African trypanosomes

**DOI:** 10.1016/j.tcsw.2023.100100

**Published:** 2023-01-28

**Authors:** Michael D. Urbaniak, Catarina Gadelha

**Affiliations:** a Division of Biomedical and Life Sciences, Faculty of Health and Medicine, Lancaster University, Lancaster LA1 4YG, United Kingdom; b School of Life Sciences, University of Nottingham, Nottingham NG7 2UH, United Kingdom

Mammals limit the availability of the essential element iron by sequestering it within the major serum glycoprotein transferrin (Tf). This is part of a strategy of immune defence against invading pathogens termed nutritional immunity. African trypanosomes have, however, evolved the means to steal iron from the mammalian bloodstream. A hallmark of the disease caused by these organisms (in particular the species *Trypanosoma brucei*, *T. congolense* and *T. vivax*) is anaemia and weight loss in a range of wild and domestic animals including cattle, sheep and goats. *T. brucei* and *T. congolense* have evolved a unique high-affinity transferrin receptor (TfR) that allows them to obtain iron through receptor-mediated endocytosis of host Tf (Steverding et al., 1995). In contrast, the *T. vivax* genome appears to lack identifiable TfR orthologues (Jackson et al., 2013). *T. brucei* is, in addition, able to sense and respond to iron starvation conditions through a yet undefined post-transcriptional mechanism important for its survival and virulence (Benz et al., 2018, Fast et al., 1999, Mußmann et al., 2003, Steverding et al., 1995), but it remains unclear if a similar mechanism is present in *T. congolense*.

In this Surface Features article, we briefly discuss what is currently known about transferrin receptor-mediated iron uptake in African trypanosomes, and raise some of the remaining unanswered questions in the field.

## 1 Regulation of the major surface gene expression in *Trypanosoma brucei* is atypical

African trypanosomes display atypical genome organisation and regulation of gene expression, with a paucity of transcriptional control. Protein coding genes, which in the great majority lack introns, are arranged in polycistronic transcription units of functionally unrelated genes that are co-transcriptionally processed by regulated 5′ *trans* splicing of capped splice-leader (SL) RNA and 3′ polyadenylation to form mature mRNA (Clayton, 2019). Further regulation of gene expression can occur through differential export from the nucleus, access to polysomes, and RNA stability through interaction with RNA binding proteins (RBPs) (Clayton, 2019).

**Fig. 1:**
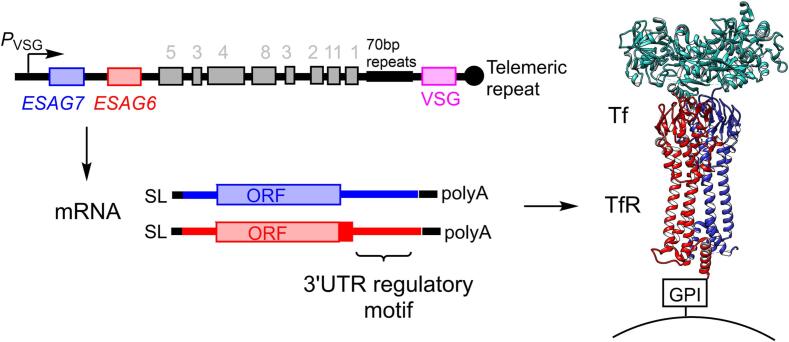
**The *Trypanosoma brucei* transferrin receptor.** The *T. brucei* TfR is encoded by the ESAG6 & ESAG7 genes on the VSG polycistronic unit within the subtelomeric expression site, with protein expression dynamically regulated via the 3′UTR. The heterodimeric receptor is attached to plasma membrane via a GPI anchor on ESAG6 (right panel, red chain) and transferrin (Tf, cyan chain) binds at an asymmetric binding site distal to the membrane. SL – spliced leader sequence, GPI – glycosylphosphatidylinositol anchor.

A subset of *T. brucei* genes are transcribed by RNA polymerase I, including the essential Variant Surface Glycoprotein (VSG). VSG forms the dense surface coat that enables the parasite to evade the host’s immune response, and undergoes antigenic variation from a repertoire of >3000 *VSG* genes (Cross et al., 2014). A single *VSG* is transcribed from only one of 15–20 subtelomeric expression sites (ESs, Fig. 1) in a specialised compartment within the nucleus. Along with this *VSG*, a set of species-specific Expression Site Associated Genes (*ESAG*s) located in the *VSG* polycistronic transcriptional unit are also transcribed (Hertz-Fowler et al., 2008). Antigenic variation requires either replacement of the *VSG* within the active ES, or a switch to a different ES – the latter also causing a change in the expressed complement of *ESAGs*. In *T. brucei*, the promoter-proximal genes *ESAG6* & *ESAG7* form the heterodimeric TfR (Steverding et al., 1995) (Fig. 1).

## 2 Major surface antigen neofunctionalization drives transferrin receptor evolution

With a vast silent repertoire of *VSG* encoded in the genome, and a mechanism of diversification through gene duplication, recombination and mosaicism, VSGs have been repurposed beyond their role as antigen variants. VSG-related proteins play roles in resistance to human serum (SRA) (De Greef, 1994), drug resistance (VSG^Sur^) (Wiedemar et al., 2018), as well as uncharacterised functions at the host-parasite interface (e.g. PAG, ESAG2, VRs) (Silva Pereira et al., 2022). TfR is also the result of neofunctionalisation of VSG. TfR and VSGs share a common ancestor (Silva Pereira et al., 2022), and still have marked structural similarity despite primary sequence divergence. Having an evolutionary origin in a variant surface antigen likely explains why the trypanosomal TfR is so distinct from its mammalian counterpart – its divergent primary sequence prevents detection by simple homology searches. And yet, structural comparisons reveal the human and trypanosome receptors to bind a similar surface of transferrin (Trevor et al., 2019). This common feature may reduce the likelihood of the host evolving a divergent transferrin surface to prevent parasite recognition – an example of co-evolutionary arms race. However, this raises the important question as to how ancestral trypanosomes scavenged iron from the mammalian host bloodstream before the evolution of a TfR.

## 3 Regulation of the *T. brucei* transferrin receptor is dynamic

Expression of TfR appears essential for survival of the mammalian lifecycle stage of *T. brucei* (Batram et al., 2014) and is dynamically regulated in response to conditions that induce iron starvation (Fast et al., 1999, Mußmann et al., 2003). Under physiological conditions, the concentration of available host Tf is unlikely to limit trypanosome growth, but Tf uptake may become limiting in later stages of infection when competition with anti-TfR antibodies and/or host anaemia come into consideration. Under iron starvation, TfR mRNA and protein levels rapidly increase, without an accompanying increase in *VSG* mRNA, suggesting that regulation occurs through an unknown post-transcriptional mechanism (Fast et al., 1999, Mußmann et al., 2003). Reducing the uptake of iron using the iron chelator deferoxamine, culturing with different mammalian sera, competition with anti-TfR antibodies or with apo-Tf all result in a rapid upregulation (∼5-fold in 6  h) of TfR and a corresponding increase in Tf uptake (Benz et al., 2018, Fast et al., 1999, Mußmann et al., 2003). Interestingly, TfR up-regulation occurs before intracellular iron stores are depleted and cells continue to divide for 48 h, suggesting that cells are responding to changes in iron flux. The dynamic regulation of the TfR is mediated through its 3′UTR, an effect independent of its genomic location in the active ES or proximity to the telomere (Benz et al., 2018). The mechanism is distinct from the post-transcriptional Iron Response Element (IRE)/Iron Response Protein (IRP) system found in mammals, as knockout of the *T. brucei* IRP-1 homologue aconitase has no effect on TfR regulation (Fast et al., 1999).

## 4 Receptor genomic location is a recent innovation in trypanosome evolution

The *T. brucei* TfR gene family encodes around 17 very similar copies of the receptor subunits, all bar one located in the subtelomeric expression sites (Fig. 1). By silencing transcription from the active ES and activating transcription on one of the silent ESs, a different *TfR* variant will be expressed. In the related species *T. congolense*, the TfR gene family shows greater diversity in sequence, with nearly 40 copies scattered in the core genome. None seem to be associated with the active *VSG*, although the lack of information on the structure of active ESs in *T. congolense* makes this difficult to interrogate fully. However, *TfR* location to the dedicated *VSG* ES appears to be a recent innovation in trypanosome evolution, and raises important questions about this large multigene family. For example, is only one *T. congolense TfR* expressed at any one time, as is in *T. brucei*, or are multiple copies of the receptor present at the protein level? If one or many, is their level modulated, and what controls this modulation? Are different receptor variants being switched, and how is this coordinated? And do all *T. congolense TfR* genes encode functional proteins with the potential to localise to the parasite surface and scavenge transferrin from the host?

The situation gets more complicated in the closely-related African trypanosome *T. vivax*, wherein transferrin receptor gene orthologues have not to date been identified. This apparent absence may be the result of the current status of available *T. vivax* genome sequence assemblies, which contain gaps and lack many of the non-core regions in which one might expect *ESAG6/7* orthologues to be found. Given similarities in genomic content and parasitic lifestyle, it is tempting to assume that, like *T. brucei* and *T. congolense*, *T. vivax* also internalise iron through uptake of host transferrin – but if the genomes genuinely lack *TfR* orthologues, they would have to do so via a different system to their close cousins.

## 5 Summary

With the availability of genome sequences and recent development of advanced genetic tools and culturing systems, previously intractable African trypanosome species are now becoming emerging models (Awuah-Mensah et al., 2021). Several studies are under way to explore their cell surface as the primary interface with the mammalian host. The transferrin receptor is one of many large surface gene families in these and related protozoa: Trans-sialidases, mucins, adenylate cyclases, amastins, invariant surface glycoproteins 65 and 75 are just a few of those. Dissecting the mechanisms that underlie the regulation of their expression may help us understand more about modulation of large surface gene families – a formidable challenge in any model system.

## CRediT authorship contribution statement

**Michael D. Urbaniak:** Conceptualization, Writing – original draft. **Catarina Gadelha:** Conceptualization, Writing – original draft.

## Declaration of Competing Interest

Given her role of Associate Editor of the journal, Catarina Gadelha had no involvement in the peer review of this article and had no access to information regarding its peer review. Full responsibility for the editorial process for this article was delegated to Neil Gow.
